# Stroke, Small‐Vessel Disease, and Occupation: Systematic Review and Data Analysis

**DOI:** 10.1161/JAHA.124.039035

**Published:** 2026-01-30

**Authors:** Thomas Zhang, Una Clancy, Ayush Singh, Stephen Makin, Caroline McHutchison, Vera Cvoro, Carmen Arteaga‐Reyes, Daniela Jaime Garcia, Will Hewins, Michael Stringer, Michael Thrippleton, Maria C. Valdes‐Hernandez, Stewart Wiseman, Francesca Chappell, Rosalind Brown, Fergus Doubal, Joanna M. Wardlaw

**Affiliations:** ^1^ Centre for Clinical Brain Sciences and UK Dementia Research Institute, University of Edinburgh Edinburgh UK; ^2^ The Centre for Rural Health, University of Aberdeen Aberdeen UK; ^3^ Division of Psychology University of Stirling Stirling UK; ^4^ Division of Psychology and Mental Health University of Manchester Manchester UK

**Keywords:** cerebral small‐vessel diseases, lacunar, occupational exposure, stroke, Cerebrovascular Disease/Stroke, Ischemic Stroke

## Abstract

**Background:**

Novel risk factors for stroke, such as occupation, are increasingly under exploration. We investigate if specific occupational exposures and settings increase the risk of developing small‐vessel disease (SVD), including SVD‐related strokes.

**Methods:**

We performed a systematic review on stroke–occupation associations and then analyzed data from patients presenting to Lothian stroke services with mild ischemic stroke (modified Rankin Scale score ≤2). We performed magnetic resonance imaging and inquired about occupational status. We assessed relationships between high‐risk occupations (per Control to Substances Hazardous to Health guidelines) and standard occupational classifications (per Standard Occupational Classifications criteria) against white matter hyperintensity volumes, SVD score, and stroke subtype.

**Results:**

Our systematic review identified 37 papers assessing occupations/broad occupational classifications (n=13), psychosocial work‐related factors (n=11), and occupational exposure to hazardous substances (n=13). We then analyzed data from 414 participants and found, after adjustment for age, hypercholesterolemia, socioeconomic status, years of education, hypertension, diabetes, and smoking history, that high‐risk occupations were associated with higher SVD scores (odds ratio, 1.64 [95% CI, 1.07–2.54]; n=357; *P*=0.02) but not for lacunar stroke subtype (odds ratio, 1.03 [95% CI, 0.64–1.67]; n=358; *P*=0.90) or white matter hyperintensity volume (% intracranial volume) (β=−0.003 [95% CI, −0.015 to 0.008]; n=357; *P*=0.60). Examples of high‐risk occupations include drivers, engineers, and skilled trade workers. No associations were found for standard occupational classifications.

**Conclusions:**

This systematic review shows limited data on stroke–occupation associations. Our analysis showed that high‐risk occupations are associated with higher SVD scores but not stroke subtype.

**Registration:**

URL: www.crd.york.ac.uk/PROSPERO; Unique Identifier: 42024466671.

**Nonstandard Abbreviations and Acronyms**
ICV: intracranial volume
MSS2: Mild Stroke Study 2
MSS3: Mild Stroke Study 3
SVD: small‐vessel disease
WMH: white matter hyperintensity

**Research Perspective**

**What Is New?**

- Our data analysis of the MSS2 (Mild Stroke Study 2) and MSS3 (Mild Stroke Study 3) cohorts identifies an association between occupational exposure to high‐risk substances, commonly found in occupations such as janitors, engineers, and machine operators, and increased cerebral small‐vessel disease burden on imaging in a stroke population.

**What Question Should Be Addressed Next?**

- Mechanistic studies are required to investigate how specific occupational exposures such as psychosocial work‐related factors and environmental toxins contribute to small‐vessel disease pathophysiology.
- Further research should confirm these findings in larger populations through prospective cohort studies with well‐defined exposures, using large‐scale epidemiological data sets to account for the potential delay between exposure and small‐vessel disease development, while also assessing how occupation interacts with traditional risk factors and other determinants of brain health, such as medication adherence and mental health.

Cerebral small‐vessel disease (SVD) affects the brain’s arterioles, venules, and capillaries and is associated with vascular dementia, gait disturbance, and disability. Magnetic resonance imaging (MRI) features of SVD include lacunes, small subcortical infarcts, white matter hyperintensities (WMHs), perivascular spaces, cerebral microbleeds, and atrophy.^1^ SVD also contributes to 25% of all strokes.^1^

While traditional stroke risk factors like vascular risk factors, socioeconomic status, and educational attainment are well known, they do not fully explain the risk for developing SVD‐related strokes.^1^ SVD‐related (ie, lacunar) stroke also has a different pathophysiology compared with non–SVD‐related stroke,^1^ so it is reasonable to explore whether alternative risk factors exist.

Midlife exposures such as occupation have been understudied to date. The interactions between stroke and occupational factors have been previously investigated, suggesting potential relationships.^2^ We are interested in further investigating all‐cause stroke because no previous systematic reviews have assessed all‐cause stroke relationships with occupation. Additionally, stroke subtyping is incomplete, which allows us to capture both ischemic strokes, including small‐vessel stroke, and hemorrhagic strokes, which may have an underlying SVD pathogenesis. Moreover, the association of occupational factors with SVD‐related stroke is unclear, as there are few studies exploring the associations between SVD‐related stroke and occupation.^2^

There are multiple occupational factors that may influence the development and progression of stroke, both SVD‐ and non–SVD‐related. Previously investigated factors such as exposure to toxic substances (carbon monoxide, carbon disulfide) have shown a high prevalence of radiological SVD features in the population.^2^ Psychosocial work factors such as workplace conflict may also impact stroke risk.^3^ We aim to investigate a range of potential occupational factors, including but not limited to toxic exposures and psychosocial work‐related factors, such as the type of occupation worked. This could potentially aid in identifying modifiable risk factors and guide interventions to reduce the burden of stroke, especially SVD‐related stroke, in the working‐age population.

To assess the knowledge base on SVD‐related stroke, non–SVD‐related stroke, and potential relationships with occupational factors, we performed a systematic review of the existing literature. Following the results of the systematic review, we also performed an analysis of patients presenting with all‐cause mild stroke to investigate potential relationships between SVD features, SVD‐related stroke, and occupation.

## Methods

### Data Availability Statement

The data, analytic methods, and study materials are available from the corresponding author upon reasonable request.

### Systematic Review

#### Search Strategy and Inclusion Criteria

We conducted the systematic review according to Preferred Reporting Items for Systematic Reviews and Meta‐Analyses guidelines, searching MEDLINE and EMBASE via OVID from inception to September 15, 2023. To investigate all‐cause stroke, on EMBASE, we used the terms *cerebrovascular disease*, *cerebrovascular accident*, *brainstem stroke*, *cardioembolic stroke*, *ischemic stroke*, *lacunar stroke*, *occupation*, *occupational exposure*, and *workplace*. On MEDLINE, we used the terms *stroke*, *lacunar*, *brain infarction*, *hemorrhagic stroke*, *ischemic stroke*, *embolic stroke*, *occupational exposure*, *occupations*, *workplace*, and *occupational diseases*. See the full search strategy in Data S1. We included primary research studies of adults in any occupation or occupationally exposed to substances who were clinically diagnosed with ischemic/hemorrhagic stroke, excluding reviews, meta‐analyses, duplicates, conference abstracts, and non–English language papers. We registered the protocol (International Prospective Register of Systematic Reviews CRD: 42024466671).

One reviewer (T.Z.) independently conducted title, abstract, and full‐text reviews of select papers using the inclusion criteria. A second reviewer (U.C.) adjudicated on a sample of papers (12%).

#### Data Extraction

Two reviewers (T.Z. and A.S.) conducted a narrative synthesis following extraction of data on population, study design, sample size, mean age, and associations between stroke and occupation with relevant effect sizes (standardized mortality ratios, adjusted hazard ratios, hazard ratios, odds ratios [ORs], subdistribution hazard ratios, and adjusted ORs). We tabulated these effect measures (Table S1). Median ages were collected when mean ages were unavailable. We stratified the data into 3 main occupational factors—the type of occupation worked/broad occupational categories, psychosocial work‐related factors, and exposure to hazardous substances—and extracted the specific factor investigated and their associations with stroke risk (eg, train drivers, conflict at work, and exposure to lead). We did not contact authors for missing information.

#### Risk‐of‐Bias Assessment

Two reviewers (T.Z. and A.S.) independently assessed bias using the Risk of Bias Assessment Tool for Nonrandomized Studies,^4^ evaluating risk as high/medium/low on the basis of participant selection, confounding variables, exposure measurement, outcome data completeness, and selective outcome reporting (Table S2). We resolved discrepancies through discussion.

### Clinical Data Analysis

#### Population

We pooled baseline cross‐sectional data from 2 prospective longitudinal observational studies: MSS2 (Mild Stroke Study 2)^5^ and MSS3 (Mild Stroke Study 3).^6^ We conducted the research according to Strengthening the Reporting of Observational Studies in Epidemiology guidelines.

#### Institutional Review Board Approval

Both studies received ethical approval from the South East Scotland Research Ethics Committee (MSS2, 09/S1101/54; MSS3, 18/SS/0044). All participants gave written informed consent.

MSS2 and MSS3 recruited 264 and 229 outpatients and inpatients with mild (nondisabling; modified Rankin Scale score ≤2) ischemic stroke from Lothian regional stroke services from 2010 to 2012^5^ and 2018 to 2021.^6^ Baseline assessments occurred within 3 months of the index stroke.^6^ All patients attended local stroke clinics, where a stroke diagnosis was made by a stroke specialist.

We excluded participants with MRI contraindications, major neurological conditions, and severe cardiac or respiratory disease.^6^ MSS2 gathered occupational data after a study protocol amendment (189/264 participants).^5^ MSS3 gathered occupational data from recruitment.^6^ All patients gave informed consent.

#### Stroke Classification From Clinical Data

A study team consisting of an expert panel of stroke physicians and neuroradiologists came to a consensus diagnosis after a review of presenting symptoms (motor or sensory deficits, hemianopia, visuospatial disorder, dysphasia, dysarthria, ataxia, and cerebellar/brainstem symptoms) and diagnostic imaging (MRI).^5^

Stroke was defined as clinical lacunar or nonlacunar ischemic stroke syndrome using a combination of clinical and imaging findings as per the Oxfordshire Community Stroke Project classification.^7^ An expert neuroradiologist (J.M.W.) assessed all MRI scans for acute ischemic lesions according to standard neuroimaging criteria. Lacunar infarcts were defined as lesions <20 mm in diameter in the deep gray or white matter of the cerebral hemispheres or brainstem.^5^ Cortical infarcts were characterized by involvement of the cortex or subcortical areas with infarcts >20 mm (striatocapsular infarcts).^5^

We recorded age, sex, and vascular risk factors including history of transient ischemic attack, stroke, smoking history, ischemic heart disease, diabetes, hypertension, atrial fibrillation, and hypercholesterolemia. We obtained lifestyle data, that is, years of education and main occupation (in free‐text format), and a Scottish Index of Multiple Deprivation score on the basis of current postcode.^8^ Participants self‐reported a response to the following question by an interviewer at the baseline within 3 months of index stroke: “What is/was your main occupation?” We derived stroke subtypes from clinical symptoms at index stroke diagnosis, supported by imaging (MRI).

#### MRI Data

Following diagnostic imaging, all participants underwent baseline MRI within 3 months of stroke using a 1.5T (MSS2) or 3T (MSS3) scanner, including T1‐weighted, T2‐weighted, fluid‐attenuated inversion recovery, diffusion‐weighted imaging sequences, susceptibility‐weighted imaging (MSS3) or T2* gradient recall echo (MSS2),^5^ and diffusion MRI.^6^ We assessed intracranial volume (ICV), cerebrospinal fluid volume, normal‐appearing white and gray matter, WMH volume, and index and prior stroke lesion volumes. We segmented intracranial and WMH volumes using a validated pipeline.^9^ A team of experienced image analysts overseen by J.M.W. derived a summary SVD score from visual ratings, according to Standards for Reporting Vascular Changes on Neuroimaging 1 criteria.^6^, ^10^, ^11^ The composite score (0–4) reflects overall SVD burden on MRI, including WMHs, lacunes, microbleeds, and perivascular spaces.^11^

#### Data Preparation

We normalized WMHs by adjusting the cubed root of WMH volume and expressing as a percentage of ICV (WMH %ICV).^12^

Two reviewers (T.Z. and U.C.) independently classified free‐text occupations per the Standard Occupational Classification 90,^13^ where jobs (eg, doctor, teacher, accountant, secretary, plumber) are grouped into 9 predefined groups: (1) managers and senior officials; (2) professionals; (3) associate professionals and technical; (4) administrative and secretarial; (5) skilled trades; (6) personal services; (7) sales and customer services; (8) process, plant, and machine operators; and (9) other occupations.

Next, we classified the free‐text occupations into high‐ or low‐risk groups. High‐risk occupations involved exposure to potentially hazardous substances (gases, fumes, dusts, vapors, industrial chemicals) as defined by Control of Substances Hazardous to Health UK criteria.^14^ This included occupations such as textile workers, factory workers, machinists, printers, photographers, engineers, builders, painters, janitors, drivers, and managers in these occupations. See Data S2 for the full list. Low‐risk jobs included administrators, civil servants, general practitioners, nurses, bankers, teachers, sales workers, and others.

Investigators were blinded to SVD score,^11^ stroke subtype, and WMH score when rating occupations. We measured Cohen’s κ to test interrater reliability. We resolved discrepant classifications through discussion.

#### Statistical Analysis

We compared 2 sets of variables (high‐/low‐risk occupations and standard occupational classifications) with 3 primary outcomes: SVD score,^11^ stroke subtype (lacunar versus cortical) and WMH %ICV.^12^ We reported values as number (percentage) or median (interquartile range).

We performed univariate analyses using the Kruskal–Wallis rank‐sum test for ordinal categorical variables (SVD score according to occupational risk/standard occupational classification groups). We used the χ^2^ test for binomial variables (stroke subtype according to occupational risk/standard occupational classification groups) and Welch’s *t* test for WMH %ICV according to occupational risk.

For multivariable analyses, we adjusted for age, hypercholesterolemia, Scottish Index of Multiple Deprivation quintile, years of education, hypertension, diabetes, and smoking history (nonsmoker versus ever‐smoker). We also checked for collinearity between these confounding factors, such as Scottish Index of Multiple Deprivation quintile and years of education, to ensure that we could include all the confounding factors without breaching the assumptions of linear regression and that our *P* values and CIs are reliable. We performed ordinal logistic regression (after checking the proportional odds assumption using Brant’s test) for SVD score versus occupational risk/standard occupational classifications. An SVD score of 0 was used as the reference category. We also performed binary logistic regression for stroke subtype versus occupational risk/standard occupational classifications, and linear regression (after checking residual plots) for WMH volume versus occupational risk/standard occupational classifications. We chose professionals as the reference category for standard occupational classifications, as they represent the highest socioeconomic group in the overall classification and have the largest sample size (n=69), providing a suitable point of comparison for analyzing differences across the other occupational groups. We report ORs, unstandardized β, and 95% CIs.

We performed statistical analyses using R version 4.1.2 (R Foundation for Statistical Computing, Vienna, Austria). MSS2 and MSS3 received approval from Lothian Ethics Medical Research Committee (REC 09/81101/54) and the Southeast Scotland Regional Ethics Committee (reference 18/SS/0044).

## Results

**Figure 1:**
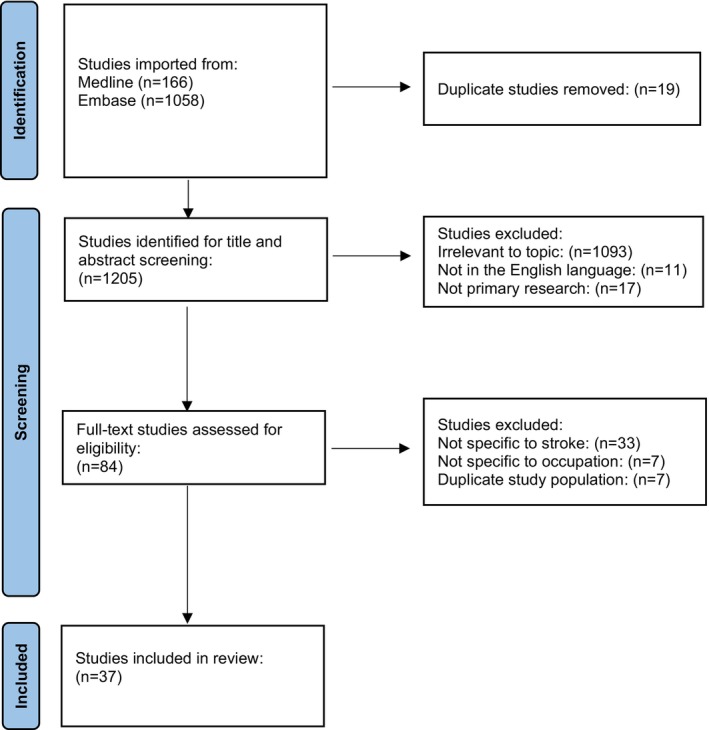
Preferred Reporting Items for Systematic Reviews and Meta‐Analyses diagram showing studies identified, screened, and included in the systematic literature review.

Our search returned 1224 papers, with full‐text review of 84 (see Preferred Reporting Items for Systematic Reviews and Meta‐Analyses flow diagram, Figure 1). We identified 37 papers studying occupation and stroke risk, reporting data on 27 477 660 patients, most (23.3 million) from a single Japanese study.^15^ The mean age was 47.3 years (n=20 studies); the median age was 44.9 years (n=2 studies); and age was unreported in n=15 studies. Included studies focused on 3 categories: (1) occupations/broad occupational classifications (n=13), (2) psychosocial work‐related factors (n=11), and (3) exposure to hazardous substances in occupational settings (n=13). We did not undertake a formal meta‐analysis due to heterogeneity in outcome measures. See Table S1 for a summary of included studies, including point estimates and 95% CIs and Figure S1 for a world map of study locations.

For specific occupations (n=7 studies, n=136 874), male Danish professional drivers (n=36 368) had an increased all‐cause stroke risk compared with men in other jobs.^16^ A Japanese study (n=32 441) found male train drivers and conductors^17^ had a lower stroke risk compared with clerical workers. Taiwanese doctors (n=28 062) had a lower stroke risk compared with the general population.^18^ No associations were found between Russian nuclear workers^19^ (n=22 377) or male iron‐ore miners^20^ (n=13 000) and stroke. Results for pig factory^21^ and seafood workers^22^ were inconclusive.

For occupational classifications (n=6 studies, n=24 412 078), a large Japanese study investigating different occupations (n=23 249 301) and industries (n=22 990 733), each coming from the same Japanese census, found that male service workers, administrators, construction workers, agriculturalists, transport and machine operators, and professional workers and engineers had a higher stroke risk compared with male sales workers.^15^ No associations were found for manufacturers, cleaners, security workers, and clerks.^15^ For industries, male employees in mining, fisheries, electricity and gas, agriculture, amusement services, hospitality, compound services, construction, transport, information, and “other” services had a higher stroke risk compared with wholesale and retail workers.^15^ A Scottish study (n=8353) found middle‐aged people of a lower occupational class (ie, manual workers) had a higher stroke risk compared with individuals of a higher occupational class.^23^ Individual studies found “overqualified women” (a discrepancy in education level and occupation) in Japan had a higher stroke risk compared with “qualified women” (n=21 599)^24^; Iranian clerical support workers had a higher stroke incidence compared with craft and trade workers (n=2440)^25^; and “blue‐collar” workers in Japan (ie, transport and machine workers) had a higher stroke risk compared with managers and professionals (n=1 128 591).^26^ No associations were found between middle‐aged Japanese factory nonmanual and manual workers (n=1794).^27^

For psychosocial work‐related factors (n=11 studies, n=325 773), a French (n=160 751) and South Korean study (n=2820) found an increased hemorrhagic stroke risk in workers with long working hours (defined as >10 hours for 50 days/year for the French study or 8–12 hours/day for the South Korean study).^28^, ^29^ A case–control study conducted on Indian workers (n=448) found sedentary occupations carried a higher ischemic stroke risk.^30^ Another case–control study found that job strain and physical/verbal conflict at work were associated with an increased stroke risk (n=198).^31^ Workplace conflict in German workers^32^ (n=7374) and US employees in long‐term protective/food preparation employment^33^ (n=13 659) were also associated with an increased stroke risk. Danish workers with increased work pressure^34^ (n=4943) and Japanese workers in high‐stress occupations^35^ (n=6553) had an increased stroke risk. Finnish workers engaging in passive commuting (n=47 721) had a higher stroke risk compared with daily active commuting (walking/cycling).^36^ Conversely, 1 study found no associations between psychological stress and stroke in Swedish male workers (n=6070).^37^ No associations were found between poor job control, high job demands, and poor social support and stroke for Swedish male construction workers (n=75 236).^38^

Numerous hazardous substances were investigated (n=13 studies, n=2 196 909). Two studies on lead exposure, 1 in American, Finnish, and British (n=88 187)^39^ and 1 in male Korean workers (n=53 970),^40^ found an increased stroke risk with high versus low lead exposure. American automobile workers exposed to metalworking fluids (n=38 553),^41^ Swedish foundry workers exposed to silica (n=2551),^42^ and British workers exposed to radiation (n=166 812)^43^ all had a higher stroke risk compared with those with no exposure. An increased stroke risk was found in Swedish manual workers exposed to particulate matters^44^ (n=983 409) and in Chinese female textile workers exposed to cotton endotoxins (n=267 400) for hemorrhagic stroke risk.^45^ Four studies (n=442 890) conducted on Scandinavian workers found no associations between noise exposure and stroke risk.^46^, ^47^, ^48^, ^49^ Inconclusive results were found for American radiological technologists exposed to radiation^50^ and American male agricultural workers exposed to pesticides.^51^

Regarding stroke subtypes, several studies investigated ischemic^28^, ^30^, ^36^, ^38^, ^41^, ^44^, ^45^ and hemorrhagic stroke,^28^, ^29^, ^45^ but none assessed small‐ versus large‐vessel strokes.

### Risk of Bias

We outline risk‐of‐bias assessment in Table S2. Nine of 37 studies had low risk of bias across all domains. The main biases included nonadjustments for confounding variables (ie, vascular risk factors).

### MSS2/MSS3 Analysis

#### Study Population and Standard Occupational Classification 90 Analyses

Table 1, which includes data from the MSS2^5^ and MSS3^6^ cohort studies, shows baseline population characteristics for 414 participants (189 from MSS2; 225 from MSS3). Seventy‐nine of 493 original participants had missing occupational data. Figure S2A shows that professional workers were the most represented (81/414 [19.6%]), whereas sales workers were the least represented (17/414 [4.3%]). Table S3 shows SVD variables: 207 of 413 (50%) patients were diagnosed with lacunar stroke. The median WMH volume was 10 (interquartile range, 4–23) mL, and the median ICV was 1549 (interquartile range, 1428–1664) mL. One hundred twenty of 413 (29%) patients had an SVD score of 0, whereas 40 of 413 (9.7%) patients had an SVD score of 4. One patient had missing SVD variables data.

**Table 1:** Baseline Clinical Population Characteristics, MSS2^5^ and MSS3^6^

	Overall, n (%) (N=414)	MSS2, n (%) N=189	MSS3, n (%) N=225
Age, median (IQR)	67 (57–75)	67 (57–75)	67 (56–74)
Sex, female	153 (37)	78 (41)	75 (33)
Transient ischemic attack	44 (11)	21 (11)	23 (10)
Stroke	48 (12)	25 (13)	23 (10)
Ischemic heart disease	65 (16)	41 (22)	24 (11)
Diabetes	70 (17)	21 (11)	49 (22)
Hypertension	292 (71)	137 (72)	155 (69)
Atrial fibrillation	33 (8.0)	13 (6.9)	20 (8.9)
Hypercholesterolemia	285 (69)	118 (62)	167 (74)
Ever‐smoker	245 (59)	119 (63)	126 (56)
Occupation			
Associate professional	28 (6.8)	11 (5.8)	17 (7.6)
Clerical	68 (16)	27 (14)	41 (18)
Craft	57 (14)	34 (18)	23 (10)
Managerial	67 (16)	27 (14)	40 (18)
Other	41 (9.9)	20 (11)	21 (9.3)
Personal and protective	18 (4.3)	2 (1.1)	16 (7.1)
Plant and machine	37 (8.9)	19 (10)	18 (8.0)
Professional	81 (20)	40 (21)	41 (18)
Sales	17 (4.1)	9 (4.8)	8 (3.6)

IQR indicates interquartile range; MSS2, Mild Stroke Study 2; and MSS3, Mild Stroke Study 3.

#### Occupational Risk and Univariate Analyses

**Figure 2:**
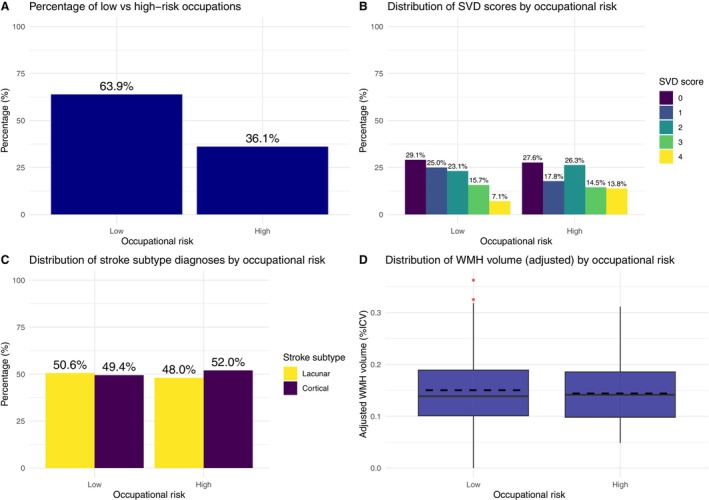
Bar charts and boxplots showing SVD scores, stroke subtype, normalized WMH volume (%ICV) and occupational risk in the Mild Stroke Studies 2 and 3. **A**, Bar chart showing the proportion of participants in low and high occupational risk groups. **B**, Grouped bar chart displaying the percentage distribution of SVD scores (0–4) across low and high occupational risk groups. **C**, Grouped bar chart showing the proportion of lacunar and cortical stroke diagnoses within low and high occupational risk groups. **D**, Boxplot showing adjusted WMH volume as a percentage of ICV, grouped by occupational risk. ICV indicates intracranial volume; SVD, small‐vessel disease; and WMH, white matter hyperintensity.

Assessing interrater reliability for classifying free‐text occupations into low‐/high‐risk occupations, we obtained a κ value (0.81). Raters agreed on 91% of classifications. Low‐risk occupations (263/413 [64%]) were more prevalent than high‐risk occupations (Figure 2A). One participant was not classified due to missing data. See Data S3 for univariate results.

#### Multivariable Analyses

For SVD score, individuals in high‐risk occupations had higher SVD scores compared with individuals in low‐risk occupations (OR, 1.64 [95% CI, 1.07–2.54]; n=357; *P*=0.02) using data from MSS2^5^ and MSS3^6^ (Table 2; Figure 2B). For standard occupational classifications, craft workers had higher increments in SVD score compared with other occupational classifications (OR, 2.94 [95% CI, 1.26–6.92]; n=353), whereas managerial workers had the lowest risk (OR,1.08 [95% CI, 0.55–2.12]; n=353; Table S4; Figure S2B).

**Table 2:** Adjusted Ordinal Logistic Regression for Associations Between SVD Scores and Occupational Risk in MSS2^5^ and MSS3^6^

	No.	OR	95% CI	*P* value
Occupational risk				0.024
Low	229	…	…	
High	128	1.64	1.07–2.54	
Age	357	1.07	1.05–1.09	<0.001
Hypercholesterolemia	357	0.87	0.56–1.34	0.5
SIMD quintile	357	0.92	0.79–1.07	0.3
Years of education	357	1.05	0.97–1.12	0.2
Hypertension	357	1.96	1.28–3.01	0.002
Diabetes	357	1.26	0.78–2.05	0.3
Smoking history				0.5
Nonsmoker	150	…	…	
Ever‐smoker	207	0.88	0.59–1.30	

MSS2 indicates Mild Stroke Study 2; MSS3, Mild Stroke Study 3; OR, odds ratio; SIMD, Scottish Index of Multiple Deprivation; and SVD, small‐vessel disease.

There was insufficient evidence to support an association between stroke subtype and occupational risk (OR, 1.03 [95% CI, 0.64–1.67]; n=358; *P*=0.90) using data from MSS2^5^ and MSS3^6^ (Table 3; Figure 2C). For standard occupational classifications, sales workers had the highest risk of lacunar (versus cortical) stroke (OR, 4.55 [95% CI, 1.00–32.8]; n=354; *P*=0.076; Table S5; Figure S2C). This estimate should be interpreted cautiously due to the small sample size (2/12) and the resulting imprecision, as reflected in the wide CI. Managerial workers had the lowest risk of lacunar stroke (OR, 0.55 [95% CI, 0.25–1.19]; n=354; *P*=0.13).

**Table 3:** Adjusted Binary Logistic Regression for Associations Between Lacunar Stroke Subtype and Occupational Risk in MSS2^5^ and MSS3^6^

	n/N	OR	95% CI	*P* value
Occupational risk				
Low	113/230	…	…	…
High	69/128	1.03	0.64–1.67	0.9
Age	182/358	0.97	0.95–0.99	0.002
Hypercholesterolemia	182/358	1.38	0.85–2.26	0.2
SIMD quintile	182/358	0.77	0.65–0.92	0.004
Total years of education	182/358	0.98	0.91–1.07	0.7
Hypertension	182/358	1.35	0.83–2.20	0.2
Diabetes	182/358	1.43	0.81–2.56	0.2
Smoking history				
Nonsmoker	77/150	…	…	…
Ever‐smoker	105/208	0.76	0.48–1.20	0.2

MSS2 indicates Mild Stroke Study 2; MSS3, Mild Stroke Study 3; OR, odds ratio; SIMD, Scottish Index of Multiple Deprivation; and SVD, small‐vessel disease.

There was insufficient evidence to support an association between occupational risk and WMH %ICV (β=−0.003 [95% CI, −0.015 to 0.008]; n=357; *P*=0.60; Table S6; Figure 2D). For standard occupational classifications, “other” workers had the highest WMH volumes (OR, 0.011 [95% CI, −0.011 to 0.033]; n=353; *P*=0.30) using data from MSS2^5^ and MSS3^6^ (Table 4; Figure S2D). Managerial workers had the lowest WMH volumes (OR, −0.011 [95% CI, −0.029 to 0.007]; n=353; *P*=0.20).

**Table 4:** Adjusted Multivariable Linear Regression for Associations Between Normalized WMH Volumes and Standard Occupational Classifications in MSS2^5^ and MSS3^6^

	No.	β	95% CI	*P* value
Occupation				
Professional	69	…	…	…
Associate professional	26	−0.008	−0.032 to 0.016	0.5
Clerical	61	−0.004	−0.022 to 0.015	0.7
Craft	40	0.004	−0.017 to 0.026	0.7
Managerial	58	−0.011	−0.029 to 0.007	0.2
Other	36	0.011	−0.011 to 0.033	0.3
Personal and protective	18	−0.008	−0.035 to 0.020	0.6
Plant and machine	33	−0.009	−0.032 to 0.014	0.5
Sales	12	0.002	−0.030 to 0.034	>0.9
Age	353	0.002	0.001–0.002	<0.001
Hypercholesterolemia	353	0.000	−0.012 to 0.012	>0.9
SIMD quintile	353	0.001	−0.003 to 0.006	0.5
Total years of education	353	−0.003	−0.005 to −0.001	0.012
Hypertension	353	0.022	0.011–0.034	<0.001
Diabetes	353	0.009	−0.005 to 0.023	0.2
Smoking history				
Nonsmoker	148	…	…	…
Ever‐smoker	205	−0.002	−0.013 to 0.009	0.7

*R* ^2^=0.279; adjusted *R* ^2^=0.247; *P*‐value≤0.001. MSS2 indicates Mild Stroke Study 2; MSS3, Mild Stroke Study 3; SIMD, Scottish Index of Multiple Deprivation; and WMH, white matter hyperintensity.

## Discussion

We found that occupational exposure to high‐risk substances (found in “high‐risk” occupations, eg, janitor or engineer) is associated with slightly higher SVD scores (Table 2; Figure 2B). We found insufficient evidence to support an association between standard occupational classifications and stroke subtype, SVD score, or WMH. Our systematic review found 37 studies (n=27 477 660) investigating associations between occupation and stroke, with many occupations, psychosocial work‐related factors, and substances investigated. However, none assessed occupational associations between small‐ versus large‐vessel stroke subtypes. Higher all‐cause stroke risk was linked to clerical support workers, professional drivers, machine operators, and manual workers. Psychosocial work‐related factors with an association with higher stroke risk included long working hours, lack of physical activity at work, and conflict at work. Substances associated with higher stroke risk include lead, metalworking fluids, silica, radiation, and cotton endotoxins. These findings from our systematic review align with our analysis of 2 studies at our center, which found an association between high‐risk occupations (drivers, machine operators, etc) and higher SVD scores. Our findings are also consistent with a study investigating occupational exposures and SVD, which found a high prevalence of radiological SVD features in people exposed to carbon monoxide/disulfide.^2^ In contrast, the mean age (47.3 years) of participants for our systematic review is much younger than other stroke cohorts due to studies investigating patients of working age.

To our knowledge, this is the first study to examine associations between occupation, stroke subtype (lacunar and cortical), and SVD features in a stroke population, unlike earlier research, which often did not specify stroke subtypes, and with only a few studies investigating ischemic^28^, ^30^, ^36^, ^38^, ^41^, ^44^, ^45^ or hemorrhagic stroke.^28^, ^29^, ^45^ No other studies investigated lacunar/cortical strokes or SVD features derived from imaging specifically. We also adjusted for multiple confounders, including vascular risk factors, educational attainment, and socioeconomic status, which are strongly linked with later‐life cerebrovascular disease and occupation.^1^ Many (19/37) papers investigated in our systematic review did not adjust for many of these factors; 18 of 37 adjusted for some.

The overall body of evidence in the existing literature is limited: Only 9 of 37 studies had low risk of bias across all domains; studies were adjusted for stroke risk factors in only half of all studies; and no study stratified results by stroke subtype. However, selective outcome reporting bias was low for all but 4 studies included in the systematic review (Table S2).

A limitation of the review process was that we searched only 2 databases: MEDLINE and EMBASE. Although these are the 2 largest biomedical databases, we acknowledge that other databases are available that address occupation. Also, we performed single‐ rather than double‐reviewer screening, but we ensured that a second reviewer adjudicated on discrepant cases. Other limitations for the analysis include the use of Standard Occupational Classifications 90 criteria,^13^ which differ from other available classifications. Furthermore, some occupational data were not acquired at the start of MSS2, and we did not collect data on participants who had multiple occupations or duration of employment. Exposure quantification was based on occupations and published occupational safety standards,^14^ but a limitation of all occupational exposure studies is that precise exposures are methodologically difficult to assess with accuracy, so our findings should be interpreted in light of this. The effect of some exposures like asbestos may have been underestimated because patients with cardiac or respiratory disease were excluded. Our analysis for standard occupational groups against stroke subtype was also limited by the small sample size and high proportion of events (10/12) for sales workers, which may affect the generalizability of the result. We considered measures such as exact logistic regression or combining the occupational classifications together, but these were unfeasible to limited data and heterogeneity between the occupational classifications. Finally, this is a secondary analysis that was not included in the original statistical analysis plan.

Several recommendations can be made for further research. Our findings should be confirmed in larger populations and through prospective cohort studies with well‐defined exposures. Large‐scale epidemiological data sets may help explore these associations, given the potential delay between exposure and SVD development. As well as the continued importance of traditional risk factors, future research models should assess the interplay of occupation with other factors (eg, medication adherence, mental health). There is also potential to investigate the mechanism of effect of occupational hazardous components on small vessels.

In conclusion, our study adds to limited existing knowledge (demonstrated by our systematic review) on relationships between occupational risk and stroke severity for SVD. Occupational exposure to high‐risk substances (for “high‐risk” occupations, eg, janitors or engineers) is associated with higher SVD scores. There is insufficient evidence to support an association between occupational risk and stroke subtype or WMH severity. Despite methodological difficulties in measuring occupational exposures, these findings provide insight into the impact of occupational factors on stroke subtypes and SVD burden.

## Sources of Funding

This work is supported by the UK Dementia Research Institute, which receives its funding from Dementia Research Institute Ltd, funded by the UK Medical Research Council, Alzheimer’s Society and Alzheimer’s Research UK (J.M.W., C.A.R., M.S.); the Fondation Leducq Network for the Study of Perivascular Spaces in Small Vessel Disease (16 CVD 05; M.S.); Stroke Association “Small Vessel Disease‐Spotlight on Symptoms” (SAPG 19\100068); the Row Fogo Charitable Trust Centre for Research into Small Vessel Diseases; Stroke Association Garfield Weston Foundation Senior Clinical Lectureship (F.N.D.) (TSALECT 2015/04); National Health Service Research Scotland (F.N.D.); Stroke Association Post‐Doctoral Fellowship (SAPDF 18/100026) (S.W.); Mexican National Council of Science and Technology (CONACYT, 2021‐000007‐01EXTF‐00234) (C.A.R.); Wellcome Trust (224912/Z/21/Z) (D.J.G.); British Heart Foundation Edinburgh Centre for Research Excellence (RE/18/5/34216); National Health Service Lothian Research and Development Office (M.J.T.); European Union Horizon 2020, PHC‐03‐15, project No666881, “SVDs@Target” (M.S.); Chief Scientist of Scotland Academic Fellowship (CAF/18/08) (U.C.); National Health Service Research Scotland Fellowship (U.C.). The MSS2 was additionally funded by the Wellcome Trust (WT088134/Z/09/A) and Chest Heart Stroke Scotland (Res14/A157). The research magnetic resonance scanners are supported by the Scottish Funding Council through the Scottish Imaging Network, A Platform for Scientific Excellence Collaboration; the 3T scanner is funded by the Wellcome Trust (104916/Z/14/Z; 088134/Z/09/A), Dunhill Trust (R380R/1114), Edinburgh and Lothians Health Foundation (2012/17), Muir Maxwell Research Fund, and the University of Edinburgh. Because this research was funded in whole or in part by Wellcome (WT088134/Z/09/A) for the purpose of open access, the author has applied a Creative Commons public copyright license to any author accepted manuscript version arising from this submission.

## Disclosures

The authors hold academic grants from government and charitable funding agencies, as outlined in the Sources of Funding.

## Supporting information

Tables S1–S6
Figures S1 and S2
Supplemental Methods

PRISMA Checklist

STROBE Checklist

## References

[1] Wardlaw JM , Smith C , Dichgans M . Mechanisms of sporadic cerebral small vessel disease: insights from neuroimaging. Lancet Neurol. 2013;12:483–497. doi: 10.1016/S1474-4422(13)70060-7 23602162 PMC3836247

[2] Clancy U , Cheng Y , Brara A , Doubal FN , Wardlaw JM . Occupational and domestic exposure associations with cerebral small vessel disease and vascular dementia: a systematic review and meta‐analysis. Alzheimers Dement. 2024;20:3021–3033. doi: 10.1002/alz.13647 38270898 PMC11032565

[3] Graber M , Baptiste L , Mohr S , Blanc‐Labarre C , Dupont G , Giroud M , Béjot Y . A review of psychosocial factors and stroke: a new public health problem. Rev Neurol. 2019;175:686–692. doi: 10.1016/j.neurol.2019.02.001 31130312

[4] Kim SY , Park JE , Lee YJ , Seo H‐J , Sheen S‐S , Hahn S , Jang B‐H , Son H‐J . Testing a tool for assessing the risk of bias for nonrandomized studies showed moderate reliability and promising validity. J Clin Epidemiol. 2013;66:408–414. doi: 10.1016/j.jclinepi.2012.09.016 23337781

[5] Makin SD , Doubal FN , Dennis MS , Wardlaw JM . Clinically confirmed stroke with negative diffusion‐weighted imaging magnetic resonance imaging: longitudinal study of clinical outcomes, stroke recurrence, and systematic review. Stroke. 2015;46:3142–3148. doi: 10.1161/STROKEAHA.115.010665 26419965 PMC4617292

[6] Clancy U , Garcia DJ , Stringer MS , Thrippleton MJ , Valdés‐Hernández MC , Wiseman S , Hamilton OKL , Chappell FM , Brown R , Blair GW , et al. Rationale and design of a longitudinal study of cerebral small vessel diseases, clinical and imaging outcomes in patients presenting with mild ischaemic stroke: Mild Stroke Study 3. Eur Stroke J. 2021;6:81–88. doi: 10.1177/2396987320929617 33817338 PMC7995323

[7] Bamford J , Sandercock P , Dennis M , Warlow C , Burn J . Classification and natural history of clinically identifiable subtypes of cerebral infarction. Lancet. 1991;337:1521–1526. doi: 10.1016/0140-6736(91)93206-o 1675378

[8] Scottish Government . Scottish index of multiple deprivation 2020. Scottish Government. Accessed April 18, 2023. https://www.gov.scot/collections/scottish‐index‐of‐multiple‐deprivation‐2020/; 2020.

[9] Valdés Hernández MC , Armitage PA , Thrippleton MJ , Chappell F , Sandeman E , Muñoz Maniega S , Shuler K , Wardlaw JM . Rationale, design and methodology of the image analysis protocol for studies of patients with cerebral small vessel disease and mild stroke. Brain Behav. 2015;5:e00415. doi: 10.1002/brb3.415 26807340 PMC4714639

[10] Duering M , Biessels GJ , Brodtmann A , Chen C , Cordonnier C , de Leeuw F‐E , Debette S , Frayne R , Jouvent E , Rost NS , et al. Neuroimaging standards for research into small vessel disease‐advances since 2013. Lancet Neurol. 2023;22:602–618. doi: 10.1016/S1474-4422(23)00131-X 37236211

[11] Staals J , Makin SDJ , Doubal FN , Dennis MS , Wardlaw JM . Stroke subtype, vascular risk factors, and total MRI brain small‐vessel disease burden. Neurology. 2014;83:1228–1234. doi: 10.1212/wnl.0000000000000837 25165388 PMC4180484

[12] Wardlaw JM , Chappell FM , Valdés Hernández MC , Makin SDJ , Staals J , Shuler K , Thrippleton MJ , Armitage PA , Muñoz‐Maniega S , Heye AK , et al. White matter hyperintensity reduction and outcomes after minor stroke. Neurology. 2017;89:1003–1010. doi: 10.1212/WNL.0000000000004328 28794252 PMC5589793

[13] Higher Education Statistics Agency . Standard occupational classification: SOC 90. Higher Education Statistics Agency. Accessed April 18, 2023. https://www.hesa.ac.uk/collection/coding‐manual‐tools/sicsocdata/soc‐90; 1990.

[14] Health and Safety Executive . EH40/2005 Workplace exposure limits: containing the list of workplace exposure limits for use with the control of substances hazardous to health regulations 2002 (as amended). Norwich: Health and Safety Executive; 2005.

[15] Wada K , Eguchi H , Prieto‐Merino D . Differences in stroke and ischemic heart disease mortality by occupation and industry among Japanese working‐aged men. SSM Popul Health. 2016;2:745–749. doi: 10.1016/j.ssmph.2016.10.004 29349185 PMC5757844

[16] Tuchsen F , Hannerz H , Roepstorff C , Krause N . Stroke among male professional drivers in Denmark, 1994‐2003. Occup Environ Med. 2006;63:456–460. doi: 10.1136/oem.2005.025718 16735481 PMC2092514

[17] Murata K , Nogawa K , Suwazono Y . The relationship between job type and development of cerebral stroke in a large, longitudinal cohort study of workers in a railway company in Japan. Atherosclerosis. 2013;229:217–221. doi: 10.1016/j.atherosclerosis.2013.04.013 23642912

[18] Tam H‐P , Lin H‐J , Weng S‐F , Hsu C‐C , Wang J‐J , Su S‐B , Huang C‐C , Guo H‐R . The risk of stroke in physicians: a population‐based cohort study in Taiwan. Epidemiology. 2017;28:S48–S53. doi: 10.1097/EDE.0000000000000720 29028675

[19] Azizova TV , Moseeva MB , Grigoryeva ES , Hamada N . Incidence risks for cerebrovascular diseases and types of stroke in a cohort of Mayak PA workers. Radiat Environ Biophys. 2022;61:5–16. doi: 10.1007/s00411-022-00966-6 35182179

[20] Bjor O , Jonsson H , Damber L , Wahlstrom J , Nilsson T . Reduced mortality rates in a cohort of long‐term underground iron‐ore miners. Am J Ind Med. 2013;56:531–540. doi: 10.1002/ajim.22168 23450695

[21] Johnson ES , Ndetan H , Felini MJ , Faramawi MF , Singh KP , Choi K‐M , Qualls‐Hampton R . Mortality in workers employed in pig abattoirs and processing plants [published correction appears in erratum in: Environ Res. 2015 Oct;142:757–9]. Environ Res. 2011;111:871–876. doi: 10.1016/j.envres.2011.06.003 21724184

[22] Johnson ES , Faramawi MF , Sall M , Choi K‐M . Cancer and noncancer mortality among American seafood workers. J Epidemiol. 2011;21:204–210. doi: 10.2188/jea.je20100147 21467730 PMC3899410

[23] Hart CL , McCartney G , Watt GCM . Occupational class differences in later life hospital use by women who survived to age 80: the Renfrew and Paisley prospective cohort study. Age Ageing. 2015;44:515–519. doi: 10.1093/ageing/afu184 25432982

[24] Honjo K , Iso H , Inoue M , Sawada N , Tsugane S . Socioeconomic status inconsistency and risk of stroke among Japanese middle‐aged women. Stroke. 2014;45:2592–2598. doi: 10.1161/strokeaha.114.005238 25028447

[25] Ghahramani R , Aghilinejad M , Kermani‐Alghoraishi M , Roohafza HR , Talaei M , Sarrafzadegan N , Sadeghi M . Occupational categories and cardiovascular diseases incidences: a cohort study in Iranian population. J Prev Med Hyg. 2020;61:e290–e295. doi: 10.15167/2421-4248/jpmh2020.61.2.1359 32803013 PMC7419113

[26] Zaitsu M , Kato S , Kim Y , Takeuchi T , Sato Y , Kobayashi Y , Kawachi I . Occupational class and risk of cardiovascular disease incidence in Japan: nationwide, multicenter, hospital‐based case‐control study. J Am Heart Assoc. 2019;8:e011350. doi: 10.1161/JAHA.118.011350 30845875 PMC6475056

[27] Li Q , Morikawa Y , Sakurai M , Nakamura K , Miura K , Ishizaki M , Kido T , Naruse Y , Suwazono Y , Nakagawa H . Occupational class and incidence rates of cardiovascular events in middle aged men in Japan. Ind Health. 2010;48:324–330. doi: 10.2486/indhealth.48.324 20562508

[28] Fadel M , Sembajwe G , Li J , Leclerc A , Pico F , Schnitzler A , Roquelaure Y , Descatha A . Association between prolonged exposure to long working hours and stroke subtypes in the CONSTANCES cohort. Occup Environ Med. 2023;80:196–201. doi: 10.1136/oemed-2022-108656 36823103

[29] Kim BJ , Lee SH , Ryu WS , Kim CK , Chung JW , Kim D , Park HK , Bae HJ , Park BJ , Yoon BW . Excessive work and risk of haemorrhagic stroke: a nationwide case‐control study. Int J Stroke. 2013;8:56–61. doi: 10.1111/j.1747-4949.2012.00949.x 23227896

[30] Kumar A , Prasad M , Kathuria P . Sitting occupations are an independent risk factor for ischemic stroke in north Indian population. Int J Neurosci. 2014;124:748–754. doi: 10.3109/00207454.2013.879130 24397501

[31] Jood K , Karlsson N , Medin J , Pessah‐Rasmussen H , Wester P , Ekberg K . The psychosocial work environment is associated with risk of stroke at working age. Scand J Work Environ Health. 2017;43:367–374. doi: 10.5271/sjweh.3636 28346817

[32] Jacob L , Kostev K . Conflicts at work are associated with a higher risk of cardiovascular disease. GMS German Med Sci. 2017;15:Doc08. doi: 10.3205/000249 PMC5406615 28496397

[33] Huynh TB , McClure LA , Howard VJ , Stafford MM , Judd SE , Burstyn I . Duration of employment within occupations and incident stroke in a US general population cohort 45 years of age or older (REGARDS study). Am J Ind Med. 2023;66:142–154. doi: 10.1002/ajim.23446 36440885

[34] Suadicani P , Andersen LL , Holtermann A , Mortensen OS , Gyntelberg F . Perceived psychological pressure at work, social class, and risk of stroke: a 30‐year follow‐up in Copenhagen male study. J Occup Environ Med. 2011;53:1388–1395. doi: 10.1097/JOM.0b013e31823c149d 22104980

[35] Tsutsumi A , Kayaba K , Kario K , Ishikawa S . Prospective study on occupational stress and risk of stroke. Arch Intern Med. 2009;169:56–61. doi: 10.1001/archinternmed.2008.503 19139324

[36] Hu G , Sarti C , Jousilahti P , Silventoinen K , Barengo NC , Tuomilehto J . Leisure time, occupational, and commuting physical activity and the risk of stroke. Stroke. 2005;36:1994–1999. doi: 10.1161/01.STR.0000177868.89946.0c 16081862

[37] Torén K , Schiöler L , Giang WK , Novak M , Söderberg M , Rosengren A . A longitudinal general population‐based study of job strain and risk for coronary heart disease and stroke in Swedish men. BMJ Open. 2014;4:e004355. doi: 10.1136/bmjopen-2013-004355 PMC3948640 24589825

[38] Schiöler L , Söderberg M , Rosengren A , Järvholm B , Torén K . Psychosocial work environment and risk of ischemic stroke and coronary heart disease: a prospective longitudinal study of 75 236 construction workers. Scand J Work Environ Health. 2015;41:280–287. doi: 10.5271/sjweh.3491 25785576

[39] Steenland K , Barry V , Anttila A , Sallmén M , McElvenny D , Todd AC , Straif K . A cohort mortality study of lead‐exposed workers in the USA, Finland and the UK. Occup Environ Med. 2017;74:785–791. doi: 10.1136/oemed-2017-104311 28546320

[40] Min Y‐S , Ahn Y‐S . The association between blood lead levels and cardiovascular diseases among lead‐exposed male workers. Scand J Work Environ Health. 2017;43:385–390. doi: 10.5271/sjweh.3631 28306758

[41] Elser H , Chen KT , Arteaga D , Reimer R , Picciotto S , Costello S , Eisen EA . Metalworking fluid exposure and stroke mortality among US autoworkers. Am J Epidemiol. 2022;191:1040–1049. doi: 10.1093/aje/kwac002 35029630 PMC9393063

[42] Fan C , Graff P , Vihlborg P , Bryngelsson I‐L , Andersson L . Silica exposure increases the risk of stroke but not myocardial infarction‐a retrospective cohort study. PLoS One. 2018;13:e0192840. doi: 10.1371/journal.pone.0192840 29481578 PMC5826533

[43] Hinksman CA , Haylock RGE , Gillies M . Cerebrovascular disease mortality after occupational radiation exposure among the UK National Registry for radiation workers cohort. Radiat Res. 2022;197:459–470. doi: 10.1667/RADE-20-00204.1 35139226

[44] Sjogren B , Lonn M , Fremling K , Feychting M , Nise G , Kauppinen T , Plato N , Wiebert P , Gustavsson P . Occupational exposure to particles and incidence of stroke. Scand J Work Environ Health. 2013;39:295–301. doi: 10.5271/sjweh.3271 22241632

[45] Gallagher LG , Ray RM , Li W , Psaty BM , Gao DL , Thomas DB , Checkoway H . Occupational exposures and mortality from cardiovascular disease among women textile workers in Shanghai, China. Am J Ind Med. 2012;55:991–999. doi: 10.1002/ajim.22113 22968969 PMC3515077

[46] Eriksson HP , Andersson E , Schiöler L , Söderberg M , Sjöström M , Rosengren A , Torén K . Longitudinal study of occupational noise exposure and joint effects with job strain and risk for coronary heart disease and stroke in Swedish men. BMJ Open. 2018;8:e019160. doi: 10.1136/bmjopen-2017-019160 PMC5892764 29615446

[47] Pettersson H , Olsson D , Järvholm B . Occupational exposure to noise and cold environment and the risk of death due to myocardial infarction and stroke. Int Arch Occup Environ Health. 2020;93:571–575. doi: 10.1007/s00420-019-01513-5 31915923 PMC7260257

[48] Stokholm ZA , Bonde JP , Christensen KL , Hansen AM , Kolstad HA . Occupational noise exposure and the risk of stroke. Stroke. 2013;44:3214–3216. doi: 10.1161/STROKEAHA.113.002798 23988647

[49] Thacher JD , Roswall N , Lissåker C , Aasvang GM , Albin M , Andersson EM , Engström G , Eriksson C , Hvidtfeldt UA , Ketzel M , et al. Occupational noise exposure and risk of incident stroke: a pooled study of five Scandinavian cohorts. Occup Environ Med. 2022;79:594–601. doi: 10.1136/oemed-2021-108053 35450950 PMC9453564

[50] Rajaraman P , Doody MM , Yu CL , Preston DL , Miller JS , Sigurdson AJ , Freedman DM , Alexander BH , Little MP , Miller DL , et al. Incidence and mortality risks for circulatory diseases in US radiologic technologists who worked with fluoroscopically guided interventional procedures, 1994‐2008. Occup Environ Med. 2016;73:21–27. doi: 10.1136/oemed-2015-102888 26350678

[51] Rinsky JL , Hoppin JA , Blair A , He K , Beane Freeman LE , Chen H . Agricultural exposures and stroke mortality in the agricultural health study. J Toxicol Environ Health A. 2013;76:798–814. doi: 10.1080/15287394.2013.819308 24028665 PMC3773612

