# Supplementary material for: Stroke, Small‐Vessel Disease, and Occupation: Systematic Review and Data Analysis
Source: J Am Heart Assoc. 2026 Jan 30;15(3):e039035. doi: 10.1161/JAHA.124.039035 (PMC13055449; doi:10.1161/JAHA.124.039035)

SUPPLEMENTAL MATERIAL

Data S1.

Embase search terms

1. cerebrovascular accident/ or cerebrovascular disease/ or brainstem stroke/ or cardioembolic stroke/ or ischemic stroke/ or lacunar stroke/
2. occupation/ or occupational exposure/ or workplace/
3. 1 and 2

Medline search terms

1. stroke/ or stroke, lacunar/ or brain infarction/ or hemorrhagic stroke.mp. or ischemic stroke/ or embolic stroke/
2. occupational exposure/ or occupations/ or workplace/ or occupational diseases/
3. 1 and 2

Data S2: Full list of high-risk occupations

Plumber
Car mechanic
Lorry driver
Railway engineer
Marine engineer
Roofer
Window fitter manager
Agricultural engineer
Courier driver/owner
Mechanical engineer
Lithographer
Railway technician
Construction worker
Transport manager
Draughtsman
Coal/oil distributor
Train driver
Fire service
Upholsterer
HGV driver
Electrical engineer
Carpet factory worker
Electronics tester
Flooring business manager
Brewer
Maintenance manager/engineer
Gas engineer
Chartered engineer
Truck mechanic
Tillage farmer
Joiner
Heating engineer
Fireman
Wire weaver
Palace driver/maintenance
Health and safety officer
Factory worker
Welder
TV service engineer
Blacksmith
Taxi driver
Janitor
Driving instructor
Industrial engineer
Papermill director
Biscuit factory worker
Bike mechanic
Civil engineer
Dry cleaning manager
Distillery worker
Electrical plumber
Transport manager
HGV mechanic
Motor mechanic
Coal mining electrician
Floor layer
Plumbing and heating director
Hospital porter
Domestic supervisor
Road worker
Delivery driver
Landscape gardener
Bus driver
Seamstress
Stone mason
Painter/decorator
Motor trader
Clothing factory machinist
Steel erector
Power station worker
Miner
Printer
Wool factory worker
Pump manufacturer
Flour miller
Photographer
Builder
Labourer
Car park attendant
Watch repairer
Book binder worker
Forklift driver
Plant operator
Overhead linesman

Data S3: Univariate analyses

In univariate analysis, there was a trend towards an association between high-risk occupations and higher SVD scores, vs low-risk occupations (Kruskal-Wallis $\chi2=2.30$, $p=0.13$); Figure 2B. There were no associations between SVD score and standard occupational classifications (Kruskal-Wallis $\chi2=5.65$, $p=0.69$); Figure S2B.

There was no association between occupational risk and stroke subtype ($\chi2=0.16$, $p=0.69$); Figure 2C. There was no association between standard occupational classifications and stroke subtype ($\chi2=12.383$, $p=0.13$); Figure S2C.

There was no association between WMH volume and occupational risk ($t=1.08$, $p=0.28$); Figure 2D. There was no association between WMH volume and standard occupational classifications (Kruskal-Wallis $\chi2=6.32$, $p=0.61$); Figure S2D.

Table S1: Summary of included studies
Occupation

Study author (year)	Population	Study design	Sample size	Mean age	Associations	Effect sizes
Azizova 2022[19]	Russian nuclear workers chronically exposed to ionising radiation	Retrospective cohort study	22,377	25.715 (combined)	Error per gray of external gamma dose for cerebrovascular disease incidence significantly decreased with increasing attained age (males and females) and duration of employment (females).<br><br>No significant associations of either stroke or its types with cumulative gamma-ray dose of external exposure or alpha-particle dose of internal exposure were found.	Error per gray was 0.37 (95% confidence interval (CI): 0.27-0.47) in males and 0.47 (95% CI: 0.31-0.66) in females for external exposure, and 0.31 (95% CI: 0.11-0.59) in males and 0.32 (95% CI: 0.11-0.61) in females for internal exposure.

Table S1: Summary of included studies
Occupation

Björ 2013[20]	Male Swedish iron-ore miners	Prospective cohort study	13,000	24.1 (at first employment)	No association was found between stroke risk and iron-miners	SMR = 0.92 (95% CI: 0.76-1.10)

Table S1: Summary of included studies
Occupation

Ghahramani 2020[25]	Iranian workers of varying occupational classes	Prospective cohort study	2440	46.97	This study indicates that working as clerical support workers (4th group of International Standard Classification of Occupations) is associated with higher stroke incidence in comparison to craft and related trades workers. In comparison to unemployed/jobless subjects, there was not a significant correlation between the nature of occupational categories and cerebrovascular event incidence.	Clerical support workers: AHR = 1.21 (95% CI: 0.72-2.04)<br><br>Adjusted for socioeconomic and metabolic variables

Table S1: Summary of included studies
Occupation

Hart 2015[23]	45–64-year-old men and women living in Renfrew and Paisley (Scottish towns)	Prospective cohort study	8353	54.4	Lower occupational class was associated with higher rate ratios for stroke compared to higher occupational classes	Social class IV-V compared with social class I: rate ratio = 1.33 (95% CI: 1.01-1.75)

Table S1: Summary of included studies
Occupation

Honjo 2014[24]	Female Japanese middle-aged workers of different occupational classes	Prospective cohort study	21,599	49.0	High academic qualifications without an appropriate job could be a risk factor for stroke among Japanese women. (overqualified vs qualified women)<br><br>Status inconsistency could be a potential explanation for the increased stroke risk among highly educated women.	Overqualified women: AHR = 2.06 (95% CI: 1.13-3.78)<br>Adjusted for age, marital status, area and education level<br><br>Highly educated manual workers: AHR = 3.47 (95% CI: 1.54-7.84)<br><br>Workers in service industry: AHR = 3.21 (95% CI: 1.49-6.90)<br><br>Both adjusted for age, marital status and geographical area

Table S1: Summary of included studies
Occupation

Johnson 2011[21]	Workers in slaughterhouses and processing plants that handle pigs, and pork butchers/meatcutters (in the US)	Retrospective cohort study	510	Not available	An 'excess occurrence' of stroke secondary to sub-arachnoid haemorrhage was found in this occupational group. These findings were based on inadequate statistical power.	Sub-arachnoid haemorrhage: SMR = 10.1 (95% CI: 1.2-36.3)
Johnson 2011[22]	American seafood workers	Retrospective cohort study	4116	Not available	This cohort had 'excess deaths' from stroke but the significance of the findings is 'unknown', owing to less than 20% of cohort being deceased	SMR = 0.5 (95% CI: 0.2-0.8))

Table S1: Summary of included studies
Occupation

Li 2010[27]	Male Japanese factory workers aged 40-59	Prospective cohort study	1,794	47.6	No significant difference in the rate of stroke risk was identified between manual and non-manual workers	Stroke risk for manual workers when compared to non-manual workers: HR = 0.97 (95% CI 0.45-2.08)<br>SMI risk in manual workers compared to non-manual workers: HR = 0.73 (95% CI: 0.3-1.79)<br><br>Cardiovascular events risk for manual workers when compared to non-manual workers: HR = 0.92 95% CI 0.53-1.61)

Table S1: Summary of included studies
Occupation

Murata 2013[17]	Male Japanese railway workers who received annual health check-ups	10-year prospective cohort study	32,441	37	Train drivers and conductors have a significantly lower risk of developing a stroke compared to clerical workers.	Train drivers: HR = 0.63 (95% CI 0.42-0.95)<br>Conductors: HR = 0.41 (95% CI, 0.24-0.71)
Tam 2017[18]	Single-specialty doctors in Taiwan	Population-based cohort study	28,062	46.8 (median)	Physicians in Taiwan were found to have a lower risk of stroke compared with the general population in this study.	AOR = 0.61 (95% CI: 0.55-0.66)<br><br>Adjusted for hypertension, diabetes mellitus, hyperlipidaemia, coronary artery disease, and active workers
Tüchsen 2006[16]	Male professional drivers living in Greater Copenhagen	Prospective cohort study	36,368	Not available	An association was found between all professional drivers and an increased risk of stroke in this study compared to ‘economically active men’	SHR = 132<br>(95% CI:<br>121-141)

Table S1: Summary of included studies
Occupation

Wada 2016[15]	Japanese men workers of varying occupations who were aged 25–59 years in 2010	Prospective cohort study	23,349,301 (occupation)<br><br>22,990,733 (industry)<br><br>Both are coming from same sample (national census)	Not available	Occupation and industry correlated with stroke.<br><br>Service and mining are high-risk for stroke.	Service workers: AHR = 4.56 (95% CI: 3.30-6.29)<br><br>Security workers: AHR = 0.99 (95% CI: 0.66-1.50)<br><br>Adjusted for age
Zaitsu 2019[26]	Japanese workers of different socio-economic classes and occupational industries	Case-control study	1,128,591	60	A lower risk of stroke was found amongst higher occupational class workers, especially managers/professionals compared to lower occupational classes.	White-collar industry, manager: AOR = 0.84 (95% CI: 0.73-0.95)<br><br>White-collar industry, professional: AOR = 0.87 (95% CI: 0.83-0.91)<br><br>Both adjusted for smoking and alcohol

Table S1: Summary of included studies
Psychosocial work-related factors

Study (year)	Population	Study design	n	Mean age	Key findings	Effect sizes
Fadel 2023[28]	French full-time workers of varying age, sex and occupation	Retrospective cohort study	160,751	Not available	Long working hours associated with increased risk of haemorrhagic stroke after adjusting for additional factors, however not associated with ischaemic stroke after adjusting for additional factors	Haemorrhagic stroke risk with Long Working Hours (LWH): AOR = 1.92 (95% CI: (1.01-3.09)<br><br>Ischaemic stroke risk with LWH: AOR = 1.01 (95% CI: 0.63-1.63)<br><br>Both adjusted for age, body mass index, high blood pressure, diabetes, dyslipidaemia, smoking and occupation

Table S1: Summary of included studies
Psychosocial work-related factors

Hu 2005[36]	Finnish male and female workers aged between 25 to 64 years without a history of CHD stroke or cancer	Prospective cohort study	47,721	Combined: 46.95	Daily active commuting compared to passive commuting is associated with reduced risk of ischaemic stroke	Low commute time: AHR = 0.93 (95% CI: 0.84-1.03)<br><br>High commute time: AHR = 0.86 (95% CI: 0.76-0.96)<br><br>Adjusted for age, sex, area, study year, BMI, SBP, cholesterol, education, smoking, alcohol consumption, diabetes, and other 2 types of physical activity

Table S1: Summary of included studies
Psychosocial work-related factors

Huynh 2023[33]	US Employed adults >= 45 Years old	Retrospective cohort study	13,659	Not available	Longer employment in in protective service, food preparation and serving and transportation and materials moving occupations may increase risk of stroke when compared to average relative risk of all occupations	Stroke risk with employment in protective services: relative risk = 2.35 (95% CI: 1.11-4.97) Stroke risk with employment in food preparation and service: relative risk = 1.55 (95% CI: 1.05-2.19) Stroke risk with employment in transportation and material moving relative risk = 1.30 (95% CI: 1.00-1.69)
Jacob 2017[32]	German Workers between ages 18 and 65 with no previous cardiovascular disease	Prospective cohort study	7374	37.5	Increased risk of stroke in workers with workplace conflicts when compared to workers without workplace conflicts	Stroke risk with workplace conflict non-adjusted OR = 1.56 (95% CI: 0.96-2.56)

Table S1: Summary of included studies
Psychosocial work-related factors

Jood 2017[31]	30–65-Year-old Swedish stroke patients with no previous history of stroke	Multicentre case control study	198	55.5	Job strain, effort reward imbalance and conflict at work were statistically significant risk factors for stroke compared to Swedish stroke-free controls	Job strain stroke risk factor (AOR = 1.30, 95% CI 1.05-1.62)<br><br>Adjusted for age, sex, job strain, conflict at work, low education, marital status, smoking, moderate/high physical activity, high blood pressure, diabetes, high blood cholesterol, body mass index, and family history of stroke.<br><br>Effort reward imbalance stroke risk factor (AOR = 1.28, 95% CI: 1.05-1.62) age, sex, ERI, conflict at work, low education, marital status, smoking, moderate/high physical activity, high blood pressure, diabetes, high blood cholesterol, body mass

Table S1: Summary of included studies
Psychosocial work-related factors

						index, and family history of stroke<br><br>Conflict at work stroke risk factor (AOR = 1.75, 95% CI 1.07-2.88)<br><br>Adjusted for age, sex, job strain, conflict at work, low education, marital status, smoking, moderate/high physical activity, high blood pressure, diabetes, high blood cholesterol, body mass index, and family history of stroke.

Table S1: Summary of included studies
Psychosocial work-related factors

Kim 2012[29]	South Koreans with no previous history of stroke or traumatic bleeding	Multicentre case control study	2820	Mean age of cases: 54.1	Blue collar workers had a higher risk of stroke than white collar workers. Long working hours was associated with increased risk of haemorrhagic stroke when compared to normal working hours.	Blue collar jobs compared with white collar jobs Stroke risk (OR = 1.33, 95% CI: 1.06- 1.66)<br>>= 13 hours a day regular working time when compared to <= 4 hours a day (OR = 1.94, 95% CI: 1.32-2.85)
Kumar 2014[30]	North Indian workers employed in ‘sitting’ occupations	Case-control study	448	53.47	The occupations which involve sitting at work were independently associated with the risk of ischemic stroke after adjustment for demographic and risk factor variables compared to North Indian controls	Occupations involving sitting at work (AOR = 2.2, 95% CI: 1.12-3.8)<br><br>Adjusted for hypertension, diabetes, dyslipidaemia, BMI, low economic status and exercise

Table S1: Summary of included studies
Psychosocial work-related factors

Schiöler 2015[38]	Male construction workers followed from 1989–2004	Prospective cohort study	75,236	59.3 (at onset)	No significant associations between poor job control, high job demands, poor social support and ischaemic stroke	Job demands HR = 1.12 (95% CI: 0.89-1.40)<br>Job control<br>HR = 1.04 (95% CI: 0.82-1.32)
Suadicani 2011[34]	Middle-aged men of varying social class in Copenhagen	Prospective cohort study	4943	48	Perceived psychological work pressure when compared to no perceived psychological work pressure is a risk factor for stroke in men of higher class. No association to stroke risk was found among low social class men.	Stroke risk with regular psychological pressure at work non-adjusted HR = 1.38 (95% CI: 1.09-1.74)
Toren 2014[37]	Swedish men born between 1915-1925 with no previous CHD or stroke history	Prospective cohort study	6070	Not available	No association was found with psychological stress at work and stroke	Low control vs high control stroke: AHR = 1.05 (95% CI: 0.89-1.24)<br><br>Adjusted for age and body mass index

Table S1: Summary of included studies
Psychosocial work-related factors

Tsutsumi 2009[35]	Japanese male and female workers	Prospective cohort study	6553	Not available	High strain, high stress occupations were associated with increased risk of stroke in Japanese men when compared to low strain, low stress jobs. No significant associations with stroke were found in women	Stroke risk in high job strain factors<br>AHR = 2.53 (95% CI: 1.08-5.94)<br><br>Adjusted for age, area, sociodemographic, smoking, alcohol, physical activity, body mass index, hypertension, diabetes mellitus, hypercholesterolaemia

Table S1: Summary of included studies
Exposure to hazardous substances

Study (year)	Population	Study design	n	Mean age	Key findings	Effect sizes
Elser 2022[41]	US Automobile workers exposed to particulate matter from metal working fluids	Retrospective and longitudinal cohort study	38,553	Not available	An association was found between a higher stroke mortality risk and metal working fluids overall ((1) middle and (2) high exposure category) and for ischemic stroke ((3) high exposure) when compared to workers with no contact to MWFs.	(1) (AHR) = 1.31, 95% (CI): 0.87-1.98)<br><br>(2) (AHR = 1.94, 95% CI: 1.13-3.16)<br><br>(3) Straight MWF (AHR = 1.45, 95% CI: 0.83-2.52) and synthetic MWF (AHR = 2.39, 95% CI: 1.39-4.50).<br><br>All adjusted for sex, race, plant location, calendar year, and year of hire

Table S1: Summary of included studies
Exposure to hazardous substances

Eriksson 2018[46]	Swedish men born in 1915–1925.	Prospective cohort study	5753	55.3	There was no significantly increased risk for stroke in any noise category (high strain vs non-high strain)	Medium noise: AHR 1.01 (95% CI: 0.84-1.21)<br><br>High noise: AHR: 1.12 (95% CI: 0.79-1.59)<br><br>Both adjusted for age, body mass index, diabetes mellitus, hypertension, cholesterol and smoking
Fan 2018[42]	Swedish foundry workers exposed to respirable silica	Retrospective cohort study	2551	Not available	Swedish foundry workers exposed to respirable silica exhibit elevated morbidity and mortality from stroke compared to the general Swedish population	(SMR 1.6, 95% CI: 1.2-2.1)

Table S1: Summary of included studies
Exposure to hazardous substances

Gallagher 2012[45]	Chinese female textile workers in Shanghai	Prospective cohort study	267,400	43 (median)	Slightly elevated mortality risk for the cotton sector was seen for (1) ischemic stroke and (2) haemorrhagic stroke compared to unexposed workers. Similar haemorrhagic stroke mortality risk was observed in high dust sectors compared to unexposed workers	(1) (AHR: 1.12, 95% CI: 0.97, 1.31)<br><br>(2) (AHR: 1.12, 95% CI: 1.02-1.23).<br><br>(3) (AHR: 1.12, 95% CI: 1.02-1.24)<br><br>Adjusted for age and smoking
Hinksman 2022[43]	British employees exposed to external radiation exposure occupationally	Prospective cohort study	166,812	Not available	The analysis has found some evidence that occupational exposure to low doses of radiation is associated with an increased risk of mortality from stroke when compared to no radiation exposure.	Error per sievert for all cerebrovascular deaths was 0.57 (95% CI: 0.00-1.31)

Table S1: Summary of included studies
Exposure to hazardous substances

Min 2017[40]	Lead-exposed Korean male workers who underwent lead-associated medical check-ups at least once between 2000 and 2004.	Prospective cohort study	53,970	37.8 (combined)	An association was found between blood lead levels and increased hospital admission for cerebrovascular disease and cerebral infarction treatment compared to low lead exposure.	AHR = 1.52 (95% CI 1.00-2.31) (BLL ≥20 µg/dL)<br><br>AHR = 2.24, 95% CI 1.14-4.39) (BLL 10–20 µg/dL)<br><br>Both adjusted for age and exposure to other metals
Pettersson 2020[47]	Workers in the Swedish construction industry that participated in health examinations between 1971 and 1993	Prospective cohort study	194,501	31	No significant association between noise exposure and stroke between high noise exposure compared to low noise exposure.	(Adjusted relative risk = 1.09 (95% CI 0.94-1.25)<br><br>Adjusted for age, body mass index, smoking habits and occupational noise

Table S1: Summary of included studies
Exposure to hazardous substances

Rajaraman 2016[50]	American radiological technologists performing fluoroscopically guided interventional procedures	Prospective cohort study	63,482	Not available	There may be elevated risk of stroke in workers performing FGIP but needs to be confirmed in studies with individual radiation dose data	Incidence HR = 1.34 (95% CI: 1.10-1.64)

Table S1: Summary of included studies
Exposure to hazardous substances

Rinsky 2013[51]	American male agricultural workers exposed to pesticides	Prospective cohort study	89,655	Not available	No measure of overall or specific pesticide use was positively associated with mortality due to stroke.<br><br>Stroke mortality was inversely associated with handling hay, grain, or silage at least once each year as reported at enrolment.<br><br>There was no evidence of an association between pesticide use and stroke mortality.	Hay, grain, silage: AHR = 0.75 (95% CI: 0.58-0.98)<br><br>Adjusted for state of residence, smoking status and alcohol consumption.

Table S1: Summary of included studies
Exposure to hazardous substances

Sjogren 2013[44]	Swedish manual workers exposed to particulate matters occupationally	Prospective cohort study	983 409	45.5 (at entrance)	Occupational exposure to small and large particles was associated with increased risks of ischaemic stroke when compared to no exposure to particles.	Men, small particles: AHR = 1.18 (95% CI: 1.09-1.27)<br><br>Men, large particles: AHR = 1.10 (95% CI: 1.02-1.19)<br><br>Women, small particles: AHR = 1.19 (95% CI: 1.03-1.38)<br><br>Women, large particles: AHR 1.12 (95% CI: 1.07-1.18)<br><br>Adjusted for age, socioeconomic groups and residential population density

Table S1: Summary of included studies
Exposure to hazardous substances

Steenland 2017[39]	American, Finnish and British workers exposed to lead occupationally	Prospective cohort mortality study	88,187	Not available	Exposure to lead is associated with a higher risk of mortality caused by stroke when compared to no exposure to lead	Blood lead levels: 20 to <30 AHR = 1.24 (95% CI: 1.03-1.50)<br><br>Blood lead levels: 30 to <40 AHR = 1.49 (95% CI: 1.20-1.85)<br><br>Blood lead levels: 40+ AHR = 1.41 (95% CI: 1.16-1.72)<br><br>Adjusted for gender, birth year decade, country.
Stokholm 2013[48]	Danish workers working either in the financial or industrial industries	Prospective cohort study	164,247	Not available	No association between occupational noise exposure and stroke for industrial workers compared to financial workers	80-84 age group: Adjusted rate ratio = 1.04 (95% CI: 0.75-1.44)<br><br>Adjusted for age, sex, socioeconomic status, calendar year, and employment status

Table S1: Summary of included studies
Exposure to hazardous substances

Thacher 2022[49]	Scandinavian workers exposed to occupational noise	Prospective pooled cohort study	78,389	55.6 (age at inclusion)	No association between occupational noise and risk of overall stroke after adjustment for confounders (high noise vs low noise jobs)	70–74 dB(A): AHR = 1.05 (0.96-1.14)<br><br>Adjustment for education level, marital status and area-income

- SMR – standardised mortality ratio
- AHR – adjusted hazard ratio
- HR – hazard ratio
- OR – odds ratio
- SHR – sub-distribution hazard ratio
- AOR – adjusted odds ratio

Table S2: Risk of bias assessment

	Selection of participants	Confounding variables	Exposure measurement adequate	Incomplete outcome data	Selective outcome reporting
Azizova 2022[19]	Low	Low	Low	Low	Low
Bjor 2013[20]	Low	High	Low	Low	Low
Ghahramani 2020[25]	Medium	Low	Low	Medium	Medium
Hart 2015[23]	Low	Medium	Low	Low	Low
Honjo 2014[24]	Medium	Low	Low	Medium	Low
Johnson 2011[21]	Medium	High	Medium	Low	Low
Johnson 2011[22]	Medium	High	Medium	Low	Low
Li 2010[27]	Low	Low	Low	Low	Low
Murata 2013[17]	Low	Low	Low	High	Medium
Tam 2017[18]	Low	Medium	Low	Low	Low
Tuchsen 2006[16]	Medium	High	Low	Low	Medium
Wada 2016[15]	Low	High	Low	Low	Low
Zaitsu 2019[26]	Low	High	Medium	Medium	Low
			Psychosocial		
	Selection of participants	Confounding variables	Exposure measurement adequate	Incomplete outcome data	Selective outcome reporting
Fadel 2023[28]	Low	Low	Low	Low	Low
Hu 2005[36]	Low	Low	Low	Low	Low
Huynh 2023[33]	Low	Medium	Low	Low	Low
Jacob 2017[32]	Low	High	Low	Low	Low
Jood 2017[31]	Low	Low	Medium	Low	Low
Kim 2012[29]	High	Low	High	High	Low
Kumar 2014[30]	Medium	Low	Medium	Low	Low
Schioler 2015[38]	Medium	Low	Low	Medium	Low
Suadicani 2011[34]	Low	Low	Low	Low	Low
Toren 2014[37]	Low	Low	Low	Low	Low
Tsutsumi 2009[35]	Low	Low	Low	Low	Low
			Substances		
	Selection of participants	Confounding variables	Exposure measurement adequate	Incomplete outcome data	Selective outcome reporting
Elser 2022[41]	Low	Medium	Low	Low	Low
Eriksson 2018[46]	Low	Low	Low	Low	Low
Fan 2018[42]	Low	High	Low	Low	Low
Gallagher 2012[45]	Low	High	Medium	Low	Medium
Hinksman 2022[43]	Low	High	Medium	Low	Low
Min 2017[40]	Low	High	Low	Low	Low
Pettersson 2020[47]	Medium	Medium	Low	High	Low
Rajaraman 2016[50]	High	Medium	Low	Medium	Low
Rinsky 2013[51]	High	Low	Low	High	Low
Sjogren 2013[44]	Medium	Low	Low	Low	Low
Steenland 2017[39]	Low	High	Medium	Low	Low
Stokholm 2013[48]	Medium	Medium	Low	Medium	Low
Thacher 2022[49]	Low	Medium	Low	Low	Low

Table S3: Small vessel disease (SVD) variables

	**Overall**, N = 413	**MSS2**, N = 189	**MSS3**, N = 224
**Final stroke subtype**	**n (%)**	**n (%)**	**n (%)**
Cortical	206 (50%)	108 (57%)	98 (44%)
Lacunar	207 (50%)	81 (43%)	126 (56%)
**SVD summary score**	**n (%)**	**n (%)**	**n (%)**
0	120 (29%)	69 (37%)	51 (23%)
1	90 (22%)	49 (26%)	41 (18%)
2	99 (24%)	39 (21%)	60 (27%)
3	64 (15%)	22 (12%)	42 (19%)
4	40 (9.7%)	10 (5.3%)	30 (13%)
**White matter hyperintensity volume (mL), median (IQR)**	10 (4, 23)	12 (4, 31)	8 (4, 19)
**Intracranial volume (mL), median (IQR)**	1,549 (1,428, 1,664)	1,485 (1,363, 1,588)	1,597 (1,493, 1,712)

Table S4: Adjusted ordinal logistic regression for SVD score associations with standard occupational classifications (Mild Stroke Study 2 and 3)

	N	OR	95% CI	p-value
**Standard occupational classifications**				0.4
Professional	69	—	—	
Associate professional	26	1.59	0.67, 3.77	
Clerical	61	1.63	0.82, 3.24	
Craft	40	2.94	1.26, 6.92	
Managerial	58	1.08	0.55, 2.12	
Other	36	1.36	0.60, 3.07	
Personal and protective	18	2.19	0.81, 5.87	
Plant and machine	33	1.70	0.75, 3.82	
Sales	12	1.34	0.41, 4.37	
Age	353	1.07	1.05-1.10	<0.001
Hypercholesterolaemia	353	0.81	0.52-1.26	0.4
SIMD quintile	353	0.91	0.78-1.06	0.2
Total years of education	353	1.08	0.99-1.17	0.076
Hypertension	353	2.08	1.35-3.23	<0.001
Diabetes	353	1.21	0.73-1.99	0.5
**Smoking history**				0.7
Non-smoker	148	—	—	
Ever-smoker	205	0.92	0.61-1.38	

Table S5: Adjusted binary logistic regression for associations between lacunar stroke subtype and occupational classification (Mild Stroke Study 2 and 3)

	n/N	OR	95% CI	p-value
**Standard occupational classifications**				
Professional	30/69	—	—	
Associate professional	14/26	0.88	0.32, 2.39	0.8
Clerical	36/62	1.41	0.64, 3.12	0.4
Craft	21/40	0.88	0.35, 2.20	0.8
Managerial	21/58	0.55	0.25, 1.19	0.13
Other	23/36	1.62	0.64, 4.19	0.3
Personal and protective	10/18	0.90	0.28, 2.93	0.9
Plant and machine	15/33	0.79	0.30, 2.06	0.6
Sales	10/12	4.55	1.00, 32.8	0.076
Age	180/354	0.97	0.95-0.99	0.006
Hypercholesterolaemia	180/354	1.37	0.83-2.28	0.2
SIMD quintile	180/354	0.76	0.63-0.91	0.003
Total years of education	180/354	1.01	0.92-1.12	0.8
Hypertension	180/354	1.41	0.85-2.34	0.2
Diabetes	180/354	1.45	0.79-2.66	0.2
**Smoking history**				
Non-smoker	75/148	—	—	
Ever-smoker	105/206	0.83	0.51-1.33	0.4

Table S6: Adjusted multivariable linear regression for associations between normalised WMH volumes and occupational risk (Mild Stroke Study 2 and 3)

	N	Beta	95% CI	p-value
**Occupational risk**				
Low	229	—	—	
High	128	-0.003	-0.015-0.008	0.6
Age	357	0.002	0.002-0.002	<0.001
Hypercholesterolaemia	357	0.000	-0.012-0.012	>0.9
SIMD quintile	357	0.001	-0.003-0.005	0.6
Years of education	357	-0.003	-0.005--0.001	0.001
Hypertension	357	0.020	0.009-0.032	<0.001
Diabetes	357	0.008	-0.006-0.022	0.3
**Smoking history**				
Non-smoker	150	—	—	
Ever-smoker	207	-0.001	-0.012-0.010	0.9
R^2=0.265; Adjusted R^2=0.248; p<0.001				

Figure S1: World map showing the locations of included studies

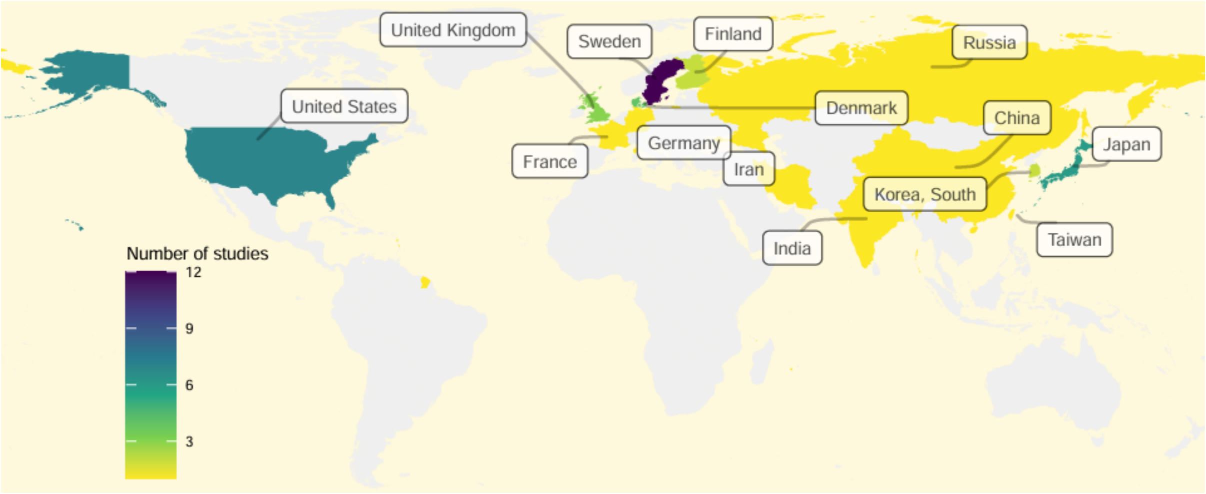

Figure S2: Bar charts and box plots showing small vessel disease (SVD) score, stroke subtype, normalised WMH (white matter hyperintensities) score (%ICV) and standard occupational classifications (Mild Stroke Studies 2/3)

(A) Bar chart showing the proportion of participants in each standard occupational classification

(B) Grouped bar chart displaying the percentage distribution of small vessel disease (SVD) scores (0–4) across standard occupational classifications

(C) Grouped bar chart showing the proportion of lacunar and cortical stroke diagnoses within standard occupational classifications

(D) Boxplot showing adjusted white matter hyperintensity (WMH) volume as a percentage of intracranial volume (%ICV), grouped by standard occupational classifications

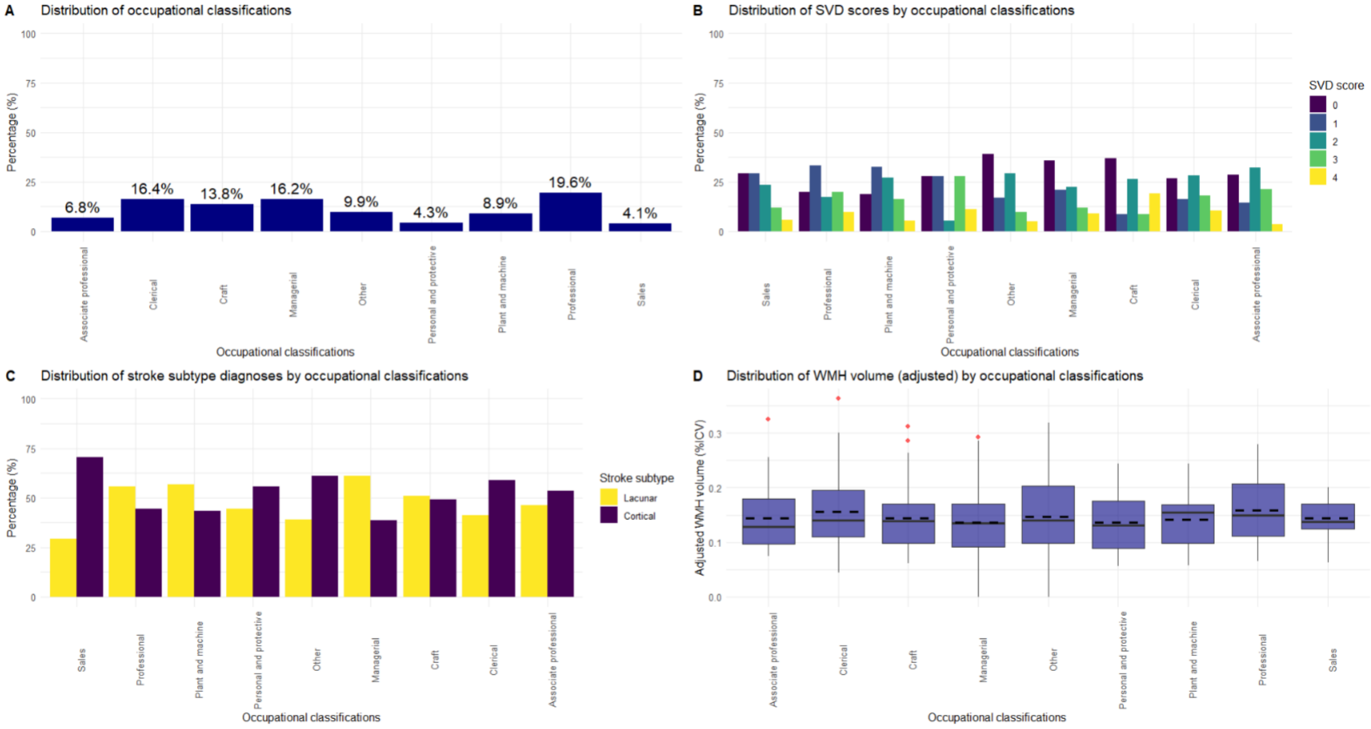

Supplement: Supplementary file 1 — Tables S1–S6 Figures S1 and S2 Supplemental Methods [file JAH3-15-e039035-s003.pdf]
